# Validity and Reproducibility of the Self-administered Food Frequency Questionnaire in the JPHC Study Cohort I: Study Design, Conduct and Participant Profiles

**DOI:** 10.2188/jea.13.1sup_2

**Published:** 2007-11-30

**Authors:** Shoichiro Tsugane, Satoshi Sasaki, Minatsu Kobayashi, Yoshitaka Tsubono, Masayuki Akabane

**Affiliations:** 1 Epidemiology and Biostatistics Division, National Cancer Center Research Institute East.; 2 Division of Epidemiology, Department of Public Health and Forensic Medicine, Tohoku University Graduate School of Medicine.; 3 Department of Nutrition, Tokyo University of Agriculture.

**Keywords:** validity, reproducibility, food frequency questionnaire, dietary record, blood, urine

## Abstract

Two kinds of food frequency questionnaire (FFQ)s were used in the baseline and 5-year follow-up survey of the Japan Public Health Center-based prospective Study on Cancer and Cardiovascular Diseases (JPHC Study Cohort I), a prospective follow-up study with 50,000 population. The former (FFQ00) was a 44-item FFQ without standard portions/units and the latter (FFQ05) was a 138-item FFQ with standard portions/units. To validate these FFQs, a 7-day dietary record (DR) survey with blood and urine collection was conducted in four seasons in the Ninohe (Iwate), Yokote (Akita) and Saku (Nagano) Public Health Center (PHC) areas, and in two seasons in the Ishikawa (Okinawa) PHC area. Another FFQ00 and FFQ05 have also been employed at a one-year interval to measure reproducibility. A total of 102 men and 113 women provided complete 28-day DRs (14 days for Okinawa) and then filled out both FFQs after three or six months. A total of 92 men and 104 women provided blood twice, and 37 men and 65 women provided 24-hour urine twice. The data from these surveys have been used to measure the validity and reproducibility of the estimated food groups and nutrient intake by each FFQ.

The accumulating evidences from large-scale prospective studies with comprehensive and validated food frequency questionnaire (FFQ) have served to disclose the role of diet in chronic disease development.^1^^,^^2^ Most of these reports were from Western countries where dietary habits and the disease profile differ substantially from those in Japan. The Japanese diet is characterized by a large number of food items per meal, various cooking methods, and seasonal variations. Therefore, the validity of the FFQ may be different from that in Western countries, making validation study necessary.

An earlier large-scale prospective study in Japan used a simple and non-validated questionnaire.^3^ It listed only 7 food items (rice/wheat, meat, fish and shellfish, milk and goat milk, green-yellow vegetables, pickles, and soybean paste soup), and questioned subjects regarding the frequency in 4 to 5 categories with the exception of rice/wheat, including the daily amount consumed. In our prospective study started in 1990, we have used two different FFQs; the FFQ in the baseline survey has 44 food items without inquires as to standard portions/units and covering 4 frequency categories, while the FFQ in a 5-year follow-up survey has 138 food items including standard portions/units and 9 frequency categories. To validate the estimated food and nutrient intake level assessed by the two FFQs, we conducted a validation study for a sub-sample of cohort participants.

In this report, we provided information on the study design, and the number of participants in the validation study. The background characteristics, food and nutrient intake among validation study participants were also compared with those among the original prospective study participants.

## METHODS

### Japan Public Health Center-based prospective Study on Cancer and Cardiovascular Diseases (JPHC Study)

The JPHC Study is a prospective follow-up study on cancer and cardiovascular diseases, initiated with 4 population cohorts and a health checkup cohort (JPHC Study Cohort I, a total of 61,595 subjects, 29,982 men and 31,613 women) in 1990.^4^ These five cohort areas were selected based on variations in the mortality rate due to stomach cancer from our previous ecological study, in which randomly selected subjects were closely examined.^5^ We further added 5 population cohorts and two Suita City cohorts (JPHC Study Cohort II, a total of 78,825 subjects, 38,740 men and 40,085 women) in 1993.^4^

This validation study was originally planned to measure the reproducibility and validity of FFQ used among participants of the JPHC Study Cohort I. However, the study was later extended to the participants of the JPHC Study Cohort II, because the same FFQ was used for their 5-year follow-up survey.^6^

#### JPHC Study Cohort I

**Figure 1.**
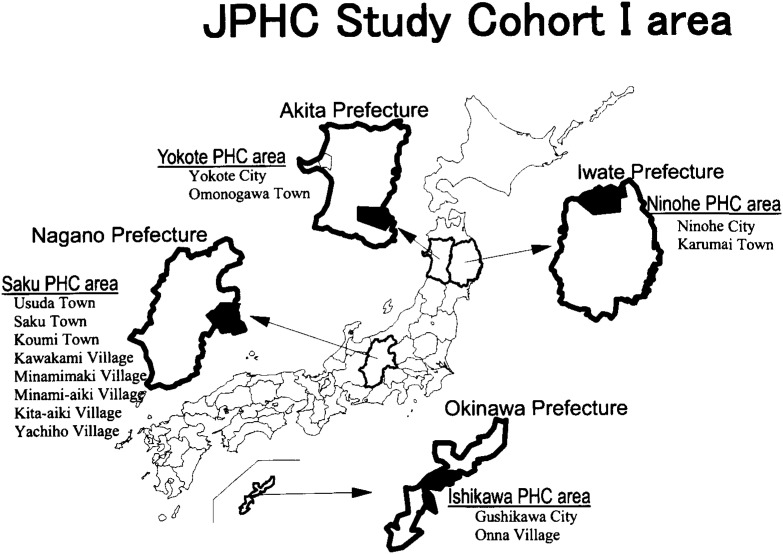
Geographic Location of the FFQ Validation study areas in the JPHC study Cohort I

As of January 1, 1990, we established a population-based cohort of 54,498 residents (27,063 men and 27,435 women) with registered addresses in 14 municipalities (city, town or village) supervised by four Public Health Centers (PHC): Ninohe PHC area (Ninohe City and Karumai Town), Iwate Prefecture; Yokote PHC area (Yokote City and Omonogawa Town), Akita Prefecture; Saku PHC areas (Usuda, Saku and Koumi Towns, the villages of Kawakami, Minamimaki, Minami-aiki, Kita-aiki and Yachiho), Nagano Prefecture; and Ishikawa PHC area (Gushikawa City and Onna Village), Okinawa Prefecture. The subjects were born between January 1, 1930 and December 31, 1949 (40 to 59 years of age) (Figure 1). We further invited 7,097 participants (2,919 men and 4,178 women) in the health checkup program in the Katshushika PHC area in the Tokyo metropolitan district from fiscal 1990 to 1994, to which all 40- to 50-year-old residents were invited. We carried out the FFQ validation study in the 4 population-based cohort areas.

#### JPHC Study Cohort II

As of January 1, 1993, we further added a population-based cohort of 62,398 residents (30,651 men and 31,747 women) with registered addresses in 13 municipalities (city, town or village) supervised by five PHCs: Mito PHC area, Ibaragi Prefecture; Kashiwazaki PHC area, Niigata Prefecture; Chuo-higashi PHC area, Kochi Prefecture; Kamigoto PHC area, Nagasaki Prefecture; and Miyako PHC area, Okinawa Prefecture. They were born between January 1, 1923 and December 31, 1952. In Suita City in the Suita PHC area, Osaka Prefecture, two different cohorts were further arranged. The first cohort (Suita 1) was defined as all 40- to 50-year-old residents (9,747, 4,793 men and 4,954 women) of Suita City as of fiscal 1993, because they were invited to the comprehensive health checkup program conducted by the city. The second cohort (Suita 2) was defined as a part of the Suita study,^7^ in which 30- to 79-year-old subjects were arbitrarily selected based on the municipal population registry, in the years 1989 through 1992, stratified by sex and arranged in 10-year age groups. The 6,680 subjects (3,296 men and 3,384 women) aged 40 to 69 years as of April 1, 1993 joined the JPHC study. Six PHC areas were selected considering geographical distribution and feasibility of the study conducted.

#### FFQs in JPHC Study Cohort I

##### FFQ in Baseline Survey (FFQ00)

In February 1990, we distributed a self-administered questionnaire which included a 44-item FFQ section (FFQ00). Questionnaires were returned from 43,149 subjects (79% response rate). The FFQ00 asked about the usual consumption of 44 food items (single item or group) during the previous month. For rice and bean paste soup, the number of bowls consumed per day was asked. For the other food items, four frequency categories were used (almost never / 1-2 days per week / 3-4 days per week / almost daily). For five alcohol beverages (sake, shochu, beer, whiskey, and other), six frequency categories were used to ask the frequency of alcohol consumption (almost never / 1-3 days per month / 1-2 days per week / 3-4 days per week / 5-6 days per week /daily). Those who consumed alcohol 1-2 days per week or more were further asked to specify the usual combinations of the beverages and the amount of each beverage consumed in a day (for instance, one bottle of beer and one bottle of sake). For 9 non-alcoholic beverages, six frequency categories were used (almost never / 1-2 days per week / 3-4 days per week / 1-2 [cups/glasses/cans/bottles] per day / 3-4 [cups/glasses/cans/bottles] per day / 5 or more [cups/glasses/cans/bottles] per day). No questions as to the standard portions/units were asked. Details of the questionnaire and the responses of the JPHC study participants were reported elsewhere.^8^ The FFQ00 is presented in the Appendix to this section.

##### FFQ in 5-year Follow-up Survey (FFQ05)

In February 1995, we conducted a 5-year follow-up survey among 52,879 residents who remained alive and were followed. A self-administered questionnaire including an 138-item FFQ section (FFQ05) was distributed. Questionnaires were returned from 42,188 subjects (77% response rate), among whom 35,945 responded to both questionnaires. The FFQ05 asked about the usual consumption of 138 food items (mostly single item or partly group items) during the previous year. Nine frequency categories were used for the majority of foods (almost never / 1-3 times per month / 1-2 times per week / 3-4 times per week / 5-6 times per week / once per day / 2-3 times per day / 4-6 times per day / 7 or more times per day). Portion sizes were specified, and the amounts were asked in three categories (less than half / same / more than one and a half times). The FFQ05 was developed based on a database of a weighed 3-day diet record survey^9^^,^^10^ from the same PHC areas in the Cohort to cover most of the population intake of most nutrients. The details of FFQ05 development were given elsewhere.^11^ The FFQ05 is presented in the Appendix to this section.

#### Validation Study Subjects

The subjects of the validation study were a subsample of the participants in the JPHC Study Cohort I. The sample size calculation was done in designing the study to detect the correlation coefficient of 0.25, which was observed in a previous study for Vitamin A with the largest within-person variation.^1^ The number of subjects required to detect this difference in correlation was approximately 112 (alpha=0.05, beta=0.20). Therefore we planned to recruit 120 pairs (120 men and 120 women) from four PHC areas, 30 pairs from each. A total of 247 subjects (122 men and 125 women, 56 from Karumai Town, Ninohe PHC area, 71 from Omonogawa Town, Yokote PHC area, 60 from Usuda and Saku Towns, Saku PHC area, and 60 from Gushikawa City and Onna Village, Ishikawa PHC area) were initially registered in the study on a voluntary basis.

#### Data Collection

**Figure 2.**
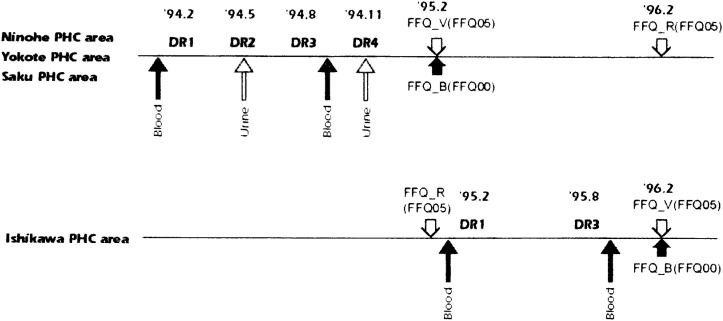
The seaqence of data collection

Figure 2 shows the sequence of data collection.

##### Diet Records (DR)

The subjects provided 7-day dietary records four times (a total of 28 days) for each of the following seasons of the year: winter (February-March) (DR1), spring (May-June) (DR2), summer (August-September) (DR3), and autumn (November-December) (DR4). In the Ishikawa PHC, where a sub-tropical climate prevails, 7-day diet records were collected only twice, Winter (DR1) and Summer (DR3), because the seasonal variation was not expected to be large.

We collected semi-weighed (weighed or standard portions/units) dietary records over seven consecutive days. Research dietitians instructed the subjects to record all foods and beverages prepared and consumed in a specially designed booklet. The participants were asked to provide detailed descriptions of each food, including the method of preparation and recipes whenever possible. The dietitians checked the records at subjects' homes, workplace or community center during the survey and reviewed them in a standardized way after completion. The mean daily consumption of energy and 16 nutrients was calculated from the records using the Standard Food Composition Table published by the Science and Technology Agency of Japan.^12^

##### Blood (BL) and Urine (UR)

A total of 25 ml blood (10 ml in heparinized tube or tube without anticoagulant) was collected by venipuncture from all participants just before (winter) or after (summer) 7-day DR. Fasting for at least 5 hours was requested before blood collection. The heparinized tube was immediately centrifuged for 10 min at 2,500-3,000 rpm to obtain plasma, red blood cell and a buffy coat layer. The tubes without anticoagulant were left for approximately one hour at room temperature to facilitate clotting, and the serum was then separated by centrifugation. The plasma, red blood cells, buffy coat layer and serum were divided into several tubes, frozen and stored in an icebox with sufficient dry ice at the examination sites until they were sent to the laboratories, where they were stored at -80°C until analysis. Plasma for ascorbic acid measurement was stabilized by metaphosphoric acid. The blood samples were sent to several laboratories for analysis of some nutrients, e.g., lipids, fatty acids, vitamins, carotenoids, and selenium.

Twenty-four hour urine samples were collected voluntarily any day during a 7-day DR. A simple portable device (Urine Mate P, Sumitomo Bakelite, Tokyo) was used for the urine collection. After measuring the total urine volume, samples were frozen at -80°C and stored until analysis of the sodium and potassium.

##### FFQs

Three months after the subjects completed the dietary records (February 1995 in the three PHC areas and February 1996 in the Ishikawa PHC area), they were requested to fill in both the FFQ00 (FFQ_B: FFQ for measuring the validity of FFQ00 used in a baseline survey) and the FFQ05 (FFQ_V: FFQ for measuring the validity of FFQ05 used in a 5-year follow-up survey). To evaluate the validìties of the FFQ00 and FFQ05, the FFQ_B and FFQ_V were administered on the occasion of a 5-year follow-up survey of JPHC Study in the three PHC areas and one year later in the Ishikawa PHC area (Figure 2).

The estimated levels of nutrient and food intake by FFQs were compared with those by dietary record or biological specimens such as blood and urine. Subjects were also asked to fill in the FFQ05 after a one-year interval to evaluate the reproducibility of FFQ05 (FFQ_R: FFQ for measuring the reproducibility of FFQ05). The FFQ_R was administered one year after the FFQ_V in the three PHC areas (February 1996) and one year before the FFQ_V in the Ishikawa PHC areas.﻿

## RESULTS

### Validation Study Participants (Table 1-2)

Among the 247 initially registered subject, 215 (87%), 102 men (84%) and 113 women (90%), 51 from Ninohe (91%), 58 from Yokote (82%), 51 from Saku (85%) and 55 from Ishikawa PHC areas (92%), provided a completed 28-day DR (14 days in the Ishikawa PHC area). All subjects who completed the DR and also answered the FFQ_V, were evaluated for the validity of these 215 FFQs against 215 completed DRs. A total of 196 subjects, 92 men and 104 women, provided blood twice, and 101 subjects, 37 men and 64 women, provided 24-hour urine twice.

**Table 1.** Male participants in validation study

	Ninohe PHC area	Yokote PHC area	Saku PHC area	Ishikawa PHC area	4 PHC areas
Initial registration	27	35	30	30	122

Questionnaire provided (FFQ)					
FFQ_V (FFQ05)	26	34	29	29	118
FFQ_R′(FFQ05)	26	32	29	28	115
FFQ_B (FFQ00)	26	34	29	29	118

Diet Records (DR)					
DR1 (Winter)	27	31	29	30	117
DR2 (Spring)	26	33	30	---	89
DR3 (Summer)	26	31	28	28	113
DR4 (Autumn)	26	32	30	---	88
28 d completed (14 d completed)	24	28	23	27	102

Blood provided (BL)					
BL1 (winter)	23	29	26	23	101
BL2 (summer)	23	26	25	23	97
Both	23	25	23	21	92
Both & 28 d	22	25	19	21	87

24-hr urine provided (UR)					
UR1 (spring)	6	24	7	---	37
UR2 (autumn)	6	30	7	---	43
Both	6	24	7	---	37
Both & 28 d	6	21	6	---	33

^1^3 areas: previous one year, Okinawa: after one year

Subject with DR, BL or UR provided at least FFQ_V.

**Table 2.** Female participants in validation study

	Ninohe PHC area	Yokote PHC area	Saku PHC area	Ishikawa PHC area	4 PHC areas
Initial registration	29	36	30	30	125

Questionnaire provided (FFQ)					
FFQ_V (FFQ05)	28	35	30	29	122
FFQ_R′(FFQ05)	27	32	29	26	114
FFQ_B (FFQ00)	28	35	30	29	122

Diet Records (DR)					
DR1 (Winter)	29	34	30	30	123
DR2 (Spring)	29	35	30	---	94
DR3 (Summer)	28	33	28	28	117
DR4 (Autumn)	28	35	30	---	93
28 d completed (14 d completed)	27	30	28	28	113

Blood provided (BL)					
BL1 (winter)	28	31	28	28	115
BL2 (summer)	27	29	25	26	107
Both	27	28	24	25	104
Both & 28 d	26	26	24	25	101

24-hr urine provided (UR)					
UR1 (spring)	18	29	20	---	67
UR2 (autumn)	18	33	18	---	69
Both	18	28	18	---	64
Both & 28 d	18	24	18	---	60

^1^3 areas: previous one year, Okinawa: after one year

Subject with DR, BL or UR provided at least FFQ_V.

### Profiles of Participants Completing DRs

The background profile of participants who completed a 28-day dietary record (14 days in Ishikawa PHC area) was shown in Table 3. The average age was 55.6 years in men and 53.3 years in women, with a mean BMI of 24.3 in men and 23.9 in women. The most frequent occupation was company employee (56%) in men, and housewife (48%) in women, followed by agricultural worker (30%) in men, and employee (26%) in women.

**Table 3.** Background characteristics of validation study participants who completed 28-day diet record (14 days in Ishikawa PHC area)

		Ninohe PHC area		Yokote PHC area		Saku PHC area		Ishikawa PHC area		4 PHC area	
				
		mean	SD	mean	SD	mean	SD	mean	SD	mean	SD
men											
n		24		28		23		27		102	
Age		55.2	5.2	55.9	4.9	58.1	5.1	53.4	4.7	55.6	5.2
Height	cm	162.9	6.0	164.6	4.8	165.4	4.6	165.0	4.1	164.5	4.9
Weight	kg	65.3	9.7	64.3	9.3	63.9	9.1	69.4	8.7	65.8	9.3
BMI		24.5	2.8	23.7	3.2	23.3	2.9	25.5	2.8	24.3	3.0

Occupation n (% of each subject)											

Agricultural work		7	(29.2)	14	(50.0)	9	(39.1)	1	(3.7)	31	(30.4)
Forestry work		2	(8.3)	0	(0)	1	(4.3)	0	(0)	3	(2.9)
Company employee		12	(50.0)	13	(46.4)	12	(52.2)	20	(74.1)	57	(55.9)
Self-employed		4	(16.7)	2	(7.1)	5	(21.7)	3	(11.1)	14	(13.7)
Professional		5	(20.8)	1	(3.6)	3	(13.0)	1	(3.7)	10	(9.8)
Unemployed		0	(0)	5	(17.9)	0	(0)	1	(3.7)	6	(5.9)
Other		1	(4.2)	2	(7.1)	1	(4.3)	1	(3.7)	5	(4.9)
Housewife											

women											
n		27		30		28		28		113	
Age		54.4	6.0	52.8	5.2	55.8	4.7	50.2	3.9	53.3	5.3
Height	cm	150.6	6.3	153.1	5.6	150.6	5.4	150.0	4.2	151.1	5.5
Weight	kg	54.7	8.3	55.3	7.6	51.9	7.1	56.2	8.7	54.6	8.0
BMI		24.0	2.6	23.6	2.9	22.9	2.8	25.0	3.8	23.9	3.1

Occupation n (% of each subject)											
Agricultural work		6	(22.2)	7	(23.3)	11	(39.3)	1	(3.6)	25	(22.1)
Forestry work		1	(3.7)	0	(0)	0	(0)	0	(0)	1	(0.9)
Company employee		6	(22.2)	11	(36.7)	3	(10.7)	9	(32.1)	29	(25.7)
Self-employed		3	(11.1)	4	(13.3)	4	(14.3)	1	(3.6)	12	(10.6)
Professional		5	(18.5)	1	(3.3)	2	(7.1)	4	(14.3)	12	(10.6)
Unemployed		0	(0)	0	(0)	1	(3.6)	0	(0)	1	(0.9)
Other		1	(3.7)	2	(6.7)	0	(0)	3	(10.7)	6	(5.3)
Housewife		10	(37.0)	15	(50.0)	17	(60.7)	12	(42.9)	54	(47.8)

## DISCUSSION

Although we should have randomly sampled the subjects of this validation study from eligible cohort participants to assure generalization of the results, we used volunteers considering the feasibility of the four 7-day DR surveys. Therefore, the validity measured in this study may not be applicable to all of the cohort participants. The background characteristics of the original JPHC Study Cohort I participants who responded to FFQ05 in the 5-year follow-up survey were shown in Table 4. Although there were slight differences in mean age, BMI and job distribution between the validation study participants and the original cohort participant, the observed difference is presumed to be appropriate in applying the validation results.

**Table 4.** Background characteristics of original cohort participants who responded to 5-year follow-up survey by PHC

		Ninohe PHC area	Yokote PHC area	Saku PHC area	Ishikawa PHC area	4 PHC areas	Excluded
men							
n		4267	5445	4856	3715	18283	
		(mean)					
Age		54.6	54.6	54.7	54.3	54.6	
Height	cm	163.1	164.2	164.3	162.3	163.6	100> or 200< 115
Weight	kg	63.2	63.0	63.l	64.9	63.4	20> or 150< 116
BMI		23.7	23.3	23.4	24.6	23.7	10> or 100< 140
Occupation		(% of each subject)					

Agricultural work		27.5	26.7	30.3	13.1	25.1	
Forestry work		2.7	0.3	2.5	0.2	1.4	
Fishing work		0.1	0.1	0.1	0.9	0.3	
Company employee		32.0	43.9	40.8	37.8	39.1	
Self-employed		16.3	196.0	20.7	14.4	18.0	
Professional		11.2	8.5	7.7	12.0	9.6	
Unemployed		6.8	8.0	4.3	9.9	7.1	
Other		10.8	5.4	4.6	10.1	7.4	
Housewife		0.1	0.1	0.1	0.3	0.1	

women							
n		5140	6573	5046	3992	20751	
		(mean)					
Age		54.8	54.9	54.9	54.1	54.7	
Height		150.6	151.9	152.0	150.3	151.3	100> or 200< 175
Weight		54.1	53.9	54.0	55.5	54.3	20> or 150< 160
BMI		23.8	23.3	23.4	24.6	23.7	10> or 100< 207
Occupation		(% of each subject)					
Agricultural work		29.9	20.6	33.8	8.7	23.9	
Forestry work		0.9	0	0.2	0.1	0.3	
Fishing work		0	0	0.1	0	0	
Company employee		23.8	21.4	30.0	22.5	24.3	
Self-employed		10.7	11.8	13.4	11.5	11.9	
Professional		5.4	3.9	4.7	6.2	4.9	
Unemployed		5.8	7.4	3.9	6.3	5.9	
Other		8.2	7.0	5.0	9.7	7.3	
Housewife		29.0	50.7	37.9	42.1	40.5	

The estimated intake of each food by FFQ05 was shown for both the validation study participants and the original JPHC Study Cohort I participants in Table 5, and the estimated intake of each nutrient was presented in Table 6. Although estimated energy intakes were almost the same in the two groups, the validation study participants tended to consume more fruit and vegetables, carotene and vitamin C. However, the average intake of other foods and nutrients was virtually comparable.

**Table 5.** Food intake estimated by FFQ05 among validation study participants and JPHC study participants who responded to FFQ05 in 5-year follow-up survey

	Validation study participants			JPHC study participants	
		
	Mean	± SD	95% CI	Mean	± SD
Men	(n=102)			(n=18,399)	
Cereals	347	± 118	(324, 371)	346	± 150
Potatoes and starches	30	± 32	(24, 37)	23	± 26
Confectioneries	16	± 22	(12, 21)	15	± 23
Fats and oils	13	± 7	(12, 15)	12	± 7
Nuts and seeds	2	± 4	(1, 3)	2	± 4
Pulses	92	± 59	(81, 104)	98	± 89
Fish and shellfish	109	± 81	(93, 125)	97	± 72
Meats	68	± 46	(59, 77)	70	± 56
Eggs	31	± 16	(28, 34)	33	± 39
Milks	199	± 235	(153, 246)	176	± 232
Vegetables	266	± 177	(231, 300)	215	± 157
Green & yellow	85	± 78	(70, 100)	62	± 57
Pickled	34	± 42	(26, 42)	28	± 35
Fruits	195	± 221	(152, 238)	132	± 142
Fungi	10	± 9	(8, 12)	9	± 10
Algae	11	± 8	(9, 13)	11	± 12
Alcoholic beverages	308	± 339	(241, 374)	310	± 381
Non-alcoholic beverages	918	± 639	(793, 1044)	828	± 588
Seasonings and spices	9	± 6	(8, 10)	8	± 6

Women		(n=113)		(n=20,922)	
Cereals	293	± 95	(275, 310)	293	± 123
Potatoes and starches	37	± 43	(29, 45)	30	± 30
Confectioneries	26	± 36	(20, 33)	22	± 27
Fats and oils	14	± 10	(12, 16)	13	± 7
Nuts and seeds	2	± 7	(1, 4)	2	± 5
Pulses	90	± 65	(78, 102)	100	± 93
Fish and shellfish	103	± 101	(84, 122)	96	± 69
Meats	56	± 39	(48, 63)	63	± 53
Eggs	29	± 17	(26, 33)	29	± 31
Milks	222	± 179	(188, 255)	215	± 241
Vegetables	297	± 251	(250, 344)	256	± 176
Green & yellow	94	± 72	(80, 107)	79	± 67
Pickled	35	± 42	(27, 43)	34	± 40
Fruits	256	± 293	(201, 310)	202	± 192
Fungi	12	± 9	(10, 14)	12	± 13
Algae	12	± 8	(11, 14)	13	± 13
Alcoholic beverages	20	± 70	(6, 33)	25	± 129
Non-alcoholic beverages	806	± 527	(708, 904)	801	± 586
Seasonings and spices	9	± 7	(8, 11)	9	± 7

**Table 6.** Nutrient intake estimated by FFQ05 among validation study participants and JPHC study participants who responded to FFQ05 in 5-year follow-up survey

	Validation study participants			JPHC study participants	
	
	Mean	± SD	95% CI	Mean	± SD
Men	(n=102)			(n=18,399)	
Energy (kcal/day)	2313	± 693	(2177, 2449)	2165	± 640
Protein (g/day)	86.8	± 35.9	(79.7, 93.8)	78.3	± 29.4
Total fat (g/day)	63.6	± 27.7	(58.2, 69)	59.0	± 27.7
carbohydrate (g/day)	304	± 100	(284, 323)	279	± 89
Alcohol (g/day)	23.9	± 23.3	(19.3, 28.4)	26.9	± 29.5
Calcium (mg/day)	656	± 393	(579, 734)	552	± 310
Phosphorus (mg/day)	1380	± 555	(1271, 1489)	1230	± 447
Iron (mg/day)	11.8	± 5.2	(10.8, 12.8)	10.3	± 4.1
Sodium (mg/day)	5615	± 2608	(5103, 6127)	5421	± 2507
Potassium (mg/day)	3212	± 1483	(2921, 3503)	2801	± 1173
Retinol (mg/day)	619	± 566	(508, 730)	602	± 676
Carotene (mg/day)	3814	± 3126	(3201, 4428)	2859	± 2551
Vitamin B1 (mg/day)	1.24	± 0.5	(1.14, 1.34)	1.16	± 0.47
Vitamin B2 (mg/day)	1.72	± 0.75	(1.58, 1.87)	1.56	± 0.70
Niacin (mg/day)	20.5	± 8.3	(18.9, 22.1)	18.7	± 7.8
Vitamin C (mg/day)	166	± 118	(143, 190)	129	± 84
Cholesterol (mg/day)	334	± 155	(304, 365)	330	± 224

Women	(n=113)			(n=20,922)	
Energy (kcal/day)	1992	± 850	(1834, 2151)	1867	± 564
Protein (g/day)	80.9	± 46.0	(72.4, 89.5)	74.9	± 28.1
Total fat (g/day)	62.8	± 36.7	(55.9, 69.6)	60.2	± 27.4
carbohydrate (g/day)	274	± 97	(256, 292)	253	± 76
Alcohol (g/day)	1.5	± 7.1	(0.2, 2.9)	1.5	± 7.5
Calcium (mg/day)	682	± 409	(606, 758)	604	± 327
Phosphorus (mg/day)	1295	± 651	(1174, 1417)	1186	± 438
Iron (mg/day)	11.8	± 7.1	(10.5, 13.2)	10.6	± 4.2
Sodium (mg/day)	5308	± 3111	(4728, 5888)	5378	± 2421
Potassium (mg/day)	3282	± 1876	(2933, 3632)	2999	± 1261
Retinol (mg/day)	592	± 697	(462, 722)	541	± 638
Carotene (mg/day)	4105	± 3029	(3540, 4670)	3623	± 2949
Vitamin B1 (mg/day)	1.21	± 0.63	(1.1, 1.33)	1.19	± 0.47
Vitamin B2 (mg/day)	1.68	± 0.86	(1.52, 1.84)	1.58	± 0.71
Niacin (mg/day)	18.0	± 10.9	(16, 20)	17.2	± 7.1
Vitamin C (mg/day)	192	± 159	(162, 222)	164	± 101
Cholesterol (mg/day)	316	± 168	(284, 347)	309	± 191

In conclusion, a total of 102 men and 113 women provided complete 28-day DRs and completed both the FFQ00 and FFQ05. Most of them provided blood twice, and a half of them also provided 24-hour urine samples twice. The data from these surveys have been used to measure the validity and reproducibility of the estimated nutrient intake by each FFQ.
